# Norepinephrine Dosage Is Associated With Lactate Clearance After Resuscitation in Patients With Septic Shock

**DOI:** 10.3389/fmed.2021.761656

**Published:** 2021-12-07

**Authors:** Chao Yu, Wenjing Fan, Min Shao

**Affiliations:** Department of Critical Care Medicine, The First Affiliated Hospital of Anhui Medical University, Hefei, China

**Keywords:** norepinephrine dosage, septic shock, vasoplegia, lactate clearance, sepsis

## Abstract

**Background:** Some septic shock patients have persistent hyperlactacidemia despite a normal systemic hemodynamics after resuscitation. Central venous oxygen saturation (ScvO_2_), mean arterial pressure (MAP), and central venous pressure (CVP) cannot be target in subsequent hemodynamic treatments. Vasoplegia is considered to be one of the main causes of oxygen metabolism abnormalities in septic shock patients, and norepinephrine (NE) is the first-line vasopressor in septic shock treatment; its dosage represents the severity of vasoplegia. This study was performed to determine whether vasoplegia, as assessed by NE dosage, can indicate patients' lactate clearance after the completion of resuscitation.

**Methods:** A retrospective study was performed, and 106 patients with septic shock in an intensive care unit were analyzed. Laboratory values and hemodynamic variables were obtained upon completion of resuscitation (H 0) and 6 h after (H 6). Lactate clearance was defined as the percent decrease in lactate from H 0 to H 6. Student's *t-*test, Mann-Whitney U-test, Chi-square or Fisher's exact tests, logistic regression analysis, and receiver operating characteristic (ROC) curve analysis were performed for statistical analysis.

**Results:** Patients with a mean age of 63.7 ± 13.8 years, baseline APACHE II score of 21.0 ± 5.1, and SOFA score of 12.7 ± 2.7 were enrolled. The study found that after 6-h of resuscitation, lactate clearance (LC) was <10% in 33 patients (31.1%). Patients with 6-h LC <10% compared with 6-h LC ≥ 10% had a higher NE dose (μg·kg^−1^·min^−1^) (0.55 [0.36–0.84] vs. 0.25 [0.18–0.41], *p* < 0.001). Multivariate logistic regression analysis of statistically significant univariate variables showed that NE dose had a significant inverse relationship with 6-h LC < 10%. The cutoff for NE was ≥ 0.32 μg·kg^−1^·min^−1^ for predicting 6-h lactate clearance after resuscitation, with a sensitivity of 75.76% and a specificity of 70.00%. Septic shock patients with an NE dose ≥ 0.32 μg·kg^−1^·min^−1^, relative to patients with an NE dose < 0.32 μg·kg^−1^·min^−1^, had a greater 30-day mortality rate (69.8% vs. 26.4% *p* < 0.001).

**Conclusion:** Some patients with septic shock had persistent oxygen metabolism disorders after hemodynamic resuscitation. NE dose may indicate vasoplegia and oxygen metabolism disorder. After resuscitation, septic shock patients with high-dose NE have lower lactate clearance and a greater 30-day mortality rate than those with low-dose NE.

## Introduction

Septic shock is defined as circulatory and cellular metabolism abnormalities induced by infection (1). Lactic acid is a sensitive indicator of anaerobic metabolism, and a number of studies have suggested that blood lactic acid levels and lactic acid clearance rates are directly related to prognosis in septic shock patients (2, 3). Even the 6-h lactate clearance rate has become one of the most important goals of septic shock resuscitation (4). For some septic shock patients with remaining hyperlactacidemia after circulation resuscitation, targets include central venous oxygen saturation (ScvO2)>70% and mean arterial pressure (MAP) > 65 mmHg (5). Hyperlactacidemia after resuscitation is still directly related to the prognosis of septic shock (6). Vasoplegia is not only one of the most important characteristics of septic shock but also an important cause of oxygen metabolism abnormalities. Norepinephrine (NE) is a confirmed first-line therapy for the treatment of vasoplegia. Recent studies have shown that early application of NE can shorten the time of resuscitation, reduce the number of resuscitation fluids needed and reduce complications including cardiogenic pulmonary edema and new-onset arrhythmia in patients with septic shock (7, 8). The relationship between NE dosage and prognosis in patients with septic shock is controversial (9, 10). However, the dosage of NE is positively correlated with the severity of vasoplegia; among monitoring indicators, NE dosage more closely reflects the hemodynamic characteristics of septic shock.

Whether NE dosage is a further indicator in patients with septic shock after circulation resuscitation is unknown. In this study, NE dosage, hemodynamic variables and clinical biochemical indexes were retrospectively analyzed as tools for forecasting lactic acid clearance after circulation resuscitation in patients with septic shock.

## Materials and Methods

### Participants and Procedures

This retrospective study was performed in the intensive care unit (ICU) of the First Affiliated Hospital of Anhui Medical University. The Institutional Research and Ethics Committee of the First Affiliated Hospital of Anhui Medical University approved this study for human subjects in accordance with the Declaration of Helsinki.

All adult patients were sequentially admitted to the ICU from August 2017 to July 2019. Patients inclusion criteria were as follows: (1) Patients fulfilled the diagnostic criteria for septic shock according to the current Third International Consensus Definitions for Sepsis and Septic Shock (Sepsis 3.0), consisting of the presence of suspected infection accompanying organ dysfunction, the use of vasopressors, MAP < 65 mmHg, and lactate levels >2 mmol/L (11). (2) All patients required central vena catheterization insertion for continuous intravenous pumping of norepinephrine, and their central venous pressure (CVP) and oxygen metabolism were monitored. (3) Patients received early resuscitation and management according to the Surviving Sepsis Campaign International Guidelines 2016 (12), with the restoration of perfusion pressure (mean arterial pressure was ≥ 65 mmHg) and perfusion flow (central venous oxygen saturation was ≥ 70%). Blood cultures were obtained from all patients before the administration of broad-spectrum antibiotics. The exclusion criteria of the study were as follows: (1) patients aged < 18 years; (2) females who were pregnant; (3) patients admitted to the ICU for < 24 h.

### Data Collection

The primary outcome variable was 6-h lactate clearance after early resuscitation.

We collected baseline characteristics, including demographic information such as age, sex and primary site of infection, the Acute Physiology and Chronic Health Evaluation II (APACHE II) score, and the Organ Failure Assessment (SOFA) score within the initial 24 h of ICU admission. Baseline vital signs (temperature, heart rate, mean arterial pressure), laboratory values and hemodynamic data were obtained after complete resuscitation (H 0), including routine blood examination, liver function, renal function, and coagulation function. Arterial and central venous blood gas were obtained at both H 0 and 6 h after resuscitation (H 6) (X-ray was used to confirm the central venous catheter position). Blood gas was tested with a bedside blood gas machine (GEM Premier 4000; Lexington, MA, USA).

### Study Definitions

6 h of Lactate Clearance Was Defined Using the Following Formula: Lactate at H 0 Minus Lactate at H 6, Divided by Lactate at H 0, and Multiplied by 100. A Positive Value Indicates a Decrease in Clearance of Lactate, Whereas a Negative Value Indicates an Increase in Lactate After 6 h.

### Statistical Analysis

We used IBM SPSS Statistics v. 19.0 (IBM, Armonk, NY, USA) for statistical analysis. The Kolmogorov–Smirnov test was used to assess the distribution of the data. Continuous variables are presented as the mean ± standard deviation ($\overset{x}{\pm }s$) for Gaussian distributions and as the median and interquartile range [M (P25, P75)] for non-Gaussian distributions. Student's t test was used for Gaussian-distributed data, and the Mann-Whitney U test was used for non-Gaussian-distributed data. The differences between categorical variables were tested by chi-square or Fisher's exact tests, if appropriate. The variables with *p* < 0.05 in univariate comparisons were then included in a multivariate logistic regression analysis of 6-h lactate clearance after resuscitation. For significant variables, receiver operating characteristic (ROC) curves were constructed to identify the predictive ability that maximized the sum of sensitivity and specificity. *p* values < 0.05 were considered statistically significant.

## Results

### Characteristics of the Patients With Septic Shock

**Figure 1:**
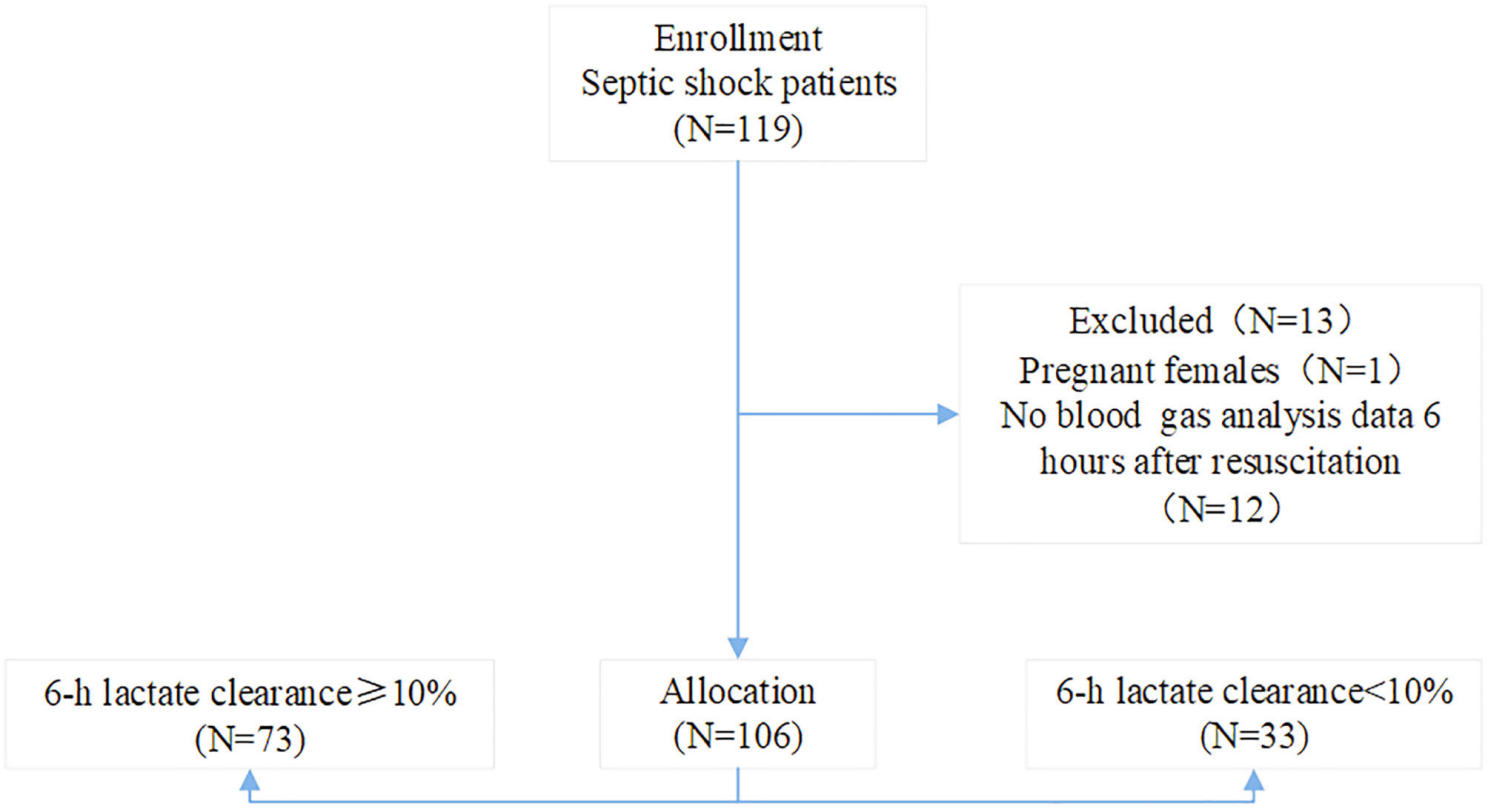
The flowchart of this study on septic shock patients.

Thirteen patients were excluded according to the exclusion criteria. A total of 106 patients, 66 male and 40 female, were enrolled from August 2017 to July 2019. The 6-h lactic acid clearance rate was still lower than 10% in some patients after resuscitation. Based on LC ≥ 10% 6 h after resuscitation, the patients were divided into the LC ≥ 10% (*n* = 73), and LC < 10% (*n* = 33) groups (Figure 1). The general characteristics and clinical data of the septic shock patients are presented in Table 1. The predominant admission diagnoses were pneumonia (37.7%) and abdominal (25.5%) infection. Patients had a mean baseline APACHE II score of 21.0 ± 5.1, and SOFA score of 12.7 ± 2.7. The 30-day in-hospital mortality rate was 48.1% (Table 1). Characteristics including age, sex, the APACHE II score and the SOFA score were not significantly different between the two groups (*P* > 0.05).

**Table 1:** Baseline characteristics and clinical data of the septic shock patients in ICU.

NO.	106
Age, years	63.7 ± 13.8
Sex (female/male)	40/66
APACHEII score	21.0 ± 5.1
SOFA score	12.7 ± 2.7
Site of infection (%)	
Lung	40 (37.7)
Abdomen	27 (25.5)
Urosepsis	19 (17.9)
Others	20 (18.9)
Heart rate (beats/min)0 h	99.7 ± 19.9
Heart rate (beats/min)6 h	98.7 ± 19.0
Temperature (°C) 0 h	37.0 (36.4–37.8)
Temperature (°C) 6 h	37.5 (36.5–38.0)
MAP (mmHg) 0 h	85.1 ± 9.2
MAP (mmHg) 6 h	84.0 ± 10.2
NE dosage (μg·kg^−1^·min^−1^) 0 h	0.31 (0.20–0.55)
NE dosage (μg·kg^−1^·min^−1^) 6 h	0.33 (0.20–0.61)
pH	7.36 ± 0.13
Base deficit, (mmol/L)	2.44 ± 6.72
Lactate (mmol/L) 0 h	4.3 (3.3–5.9)
Lactate (mmol/L) 6 h	3.6 (2.2–4.9)
CVP (mmHg) 0 h	9.4 ± 2.2
CVP (mmHg) 6 h	9.0 ± 2.1
Pv-aCO_2_ (mmHg) 0 h	6.0 (4.1–8.8)
ScvO_2_ (%)0 h	76.1 ± 4.3
6 h Lactate Clearance (%)	0.17 ± 0.29
Creatinine (mmol/L)	103.5 (75.0–159.8)
D-Dimer (ug/mL)	2.36 (0.86–3.66)
Prothrombin Time (secs)	16.3 (14.3–19.6)
Total bilirubin (mmol/L)	24.1 (16.3–36.4)
Platelet, (per mm^3^)	153,000 (94,250–214,750)
Albumin, g/L	29.7 ± 6.1
WBC (per mm^3^)	15.1 ± 5.6
30-day mortality rate (%)	48.1

*Date were presented by mean ± SD or median (P25,P75); LC, Lactate Clearance; APACHE II, Acute physiology and chronic health II; SOFA, Sequential Organ Failure Assessment; MAP, mean arterial pressure; CVP, central venous pressure; ScvO_2_, central venous oxygen saturation; Pv-aCO_2_, Central Venous-to-Arterial CO2 Difference; WBC, white blood cell*.

### Hemodynamics and Metabolic Variables in the Different Lactate Clearance Groups at Intensive Care Unit Admission

Univariate comparisons of age, sex, APACHE II score, SOFA score, vital signs, laboratory values, hemodynamics, and metabolic variables between the LC ≥ 10% and LC < 10% groups were performed (Table 2). There was no significant difference in lactate level at H 0, and at H 6, the lactate level of the LC ≥ 10% group was significantly lower than that of the LC < 10% group (*p* < 0.001). There were statistically significant differences between the two groups at H 0 in albumin, total bilirubin, lactate, and NE dosage (all *p* < 0.05). Multivariate logistic regression modeling was then performed using the variables that were statistically significant in the univariate model. Only NE dosage was significantly associated with 6-h lactate clearance < 10% in the multivariate comparison on (95% CI: 5.399–207.617) (*p* < 0.001) (Table 3).

**Table 2:** Univariate comparisons between LC ≥ 10% and LC < 10% groups.

**Factors**	**LC ≥ 10%** **(*n* = 73)**	**LC < 10%** **(*n* = 33)**	***P* Value**
Age, years	64.0 ± 13.5	62.9 ± 14.5	0.12
Sex (female/male)	28/45	12/21	0.85
APACHEII score	20.5 ± 4.9	22.2 ± 5.6	0.71
SOFA score	12.6 ± 2.7	12.8 ± 2.8	0.80
Heart rate (beats/min) 0 h	98.9 ± 18.9	101.5 ± 22.0	0.54
Heart rate (beats/min) 6 h	97.8 ± 18.0	100.7 ± 21.3	0.47
Temperature (°C) 0 h	37.1 (36.4–37.8)	37 (36.4–37.6)	0.35
Temperature (°C) 6 h	37.1 (36.6–38.0)	37 (36.5–38.0)	0.44
MAP (mmHg) 0 h	85.9 ± 9.4	83.4 ± 8.5	0.19
MAP (mmHg) 6 h	86.2 ± 10.0	79.2 ± 9.2	0.001^*^
NE dosage (μg·kg^−1^·min^−1^) 0 h	0.25 (0.18–0.41)	0.55 (0.36–0.84)	0.001^*^
NE dosage (μg·kg^−1^·min^−1^) 6 h	0.25 (0.18–0.44)	0.62 (0.32–0.88)	0.001^*^
pH 0 h	7.35 ± 0.14	7.39 ± 0.12	0.10
pH 6 h	7.37 ± 0.11	7.38 ± 0.10	0.50
Base deficit, (mmol/L)0 h	2.03 ± 6.84	3.34 ± 6.45	0.35
Base deficit, (mmol/L)6 h	2.80 ± 4.45	2.62 ± 5.96	0.87
Lactate (mmol/L) 0 h	4.2 (3.3–5.5)	4.4 (3.0–7.1)	0.70
Lactate (mmol/L) 6 h	3.0 (2.1–4.2)	4.6 (3.8–7.2)	0.001^*^
CVP (mmHg) 0 h	9.4 ± 2.2	9.5 ± 2.1	0.83
CVP (mmHg) 6 h	9.2 ± 2.2	8.7 ± 1.7	0.23
Pv-aCO_2_ (mmHg) 0 h	6.0 (4.1–8.2)	6.9 (4.3–10.0)	0.10
ScvO_2_ (%)0 h	76.1 ± 4.0	76.3 ± 5.0	0.76
6 h Lactate Clearance (%)	0.31 ± 0.15	−0.15 ± 0.20	0.001^*^
Hemoglobin (g/dL)	8.78 ± 1.70	8.39 ± 1.50	0.24
Creatinine (mmol/L)	105 (74.0–161.5)	102.0 (77.0–171.0)	0.92
Urine output 6 h (ml)	440 (260–640)	420 (262.5–580)	0.29
D-Dimer (ug/mL)	2.36 (0.98–3.65)	2.60 (0.61–5.15)	0.97
Prothrombin Time (secs)	16.3 (14.2–19.3)	16.6 (15.0–20.1)	0.24
Total bilirubin (mmol/L)	22.9 (15.5–33.8)	30.6 (16.7–42.4)	0.02
Platelet, (per mm^3^)	156,000 (102,000–216,500)	106,000 (77,500–216,500)	0.13
Albumin, g/L	30.5 ± 6.3	27.7 ± 5.3	0.03^*^
WBC (per mm^3^)	14.7 ± 5.3	15.8 ± 6.2	0.37
30-day mortality rate (%)	43.8	57.6	0.19

*Date were presented by mean ± SD or median (P25,P75); LC, Lactate Clearance; APACHE II, Acute physiology and chronic health II; SOFA, Sequential Organ Failure Assessment; MAP, mean arterial pressure; CVP, central venous pressure; ScvO_2_, central venous oxygen saturation; Pv-aCO_2_, Central Venous-to-Arterial CO2 Difference; WBC, white blood cell*.

* *Statistically significant, p <0.05*.

**Table 3:** Multivariate logistic regression modeling using statistically significant univariate variables associated with 6 h Lactate Clearance < 10%.

**Variable**	**P Value**	**OR**	**95% CI for OR**	
			**Lower**	**Upper**
Albumin	0.172	1.000	0.869	1.025
Total bilirubin	0.939	0.944	0.995	1.005
NE dosage	0.001	33.482	5.399	207.617
APACHEII score	0.179	1.068	0.970	1.176

### NE Dosage as a Predictor of Lactate Clearance

**Figure 2:**
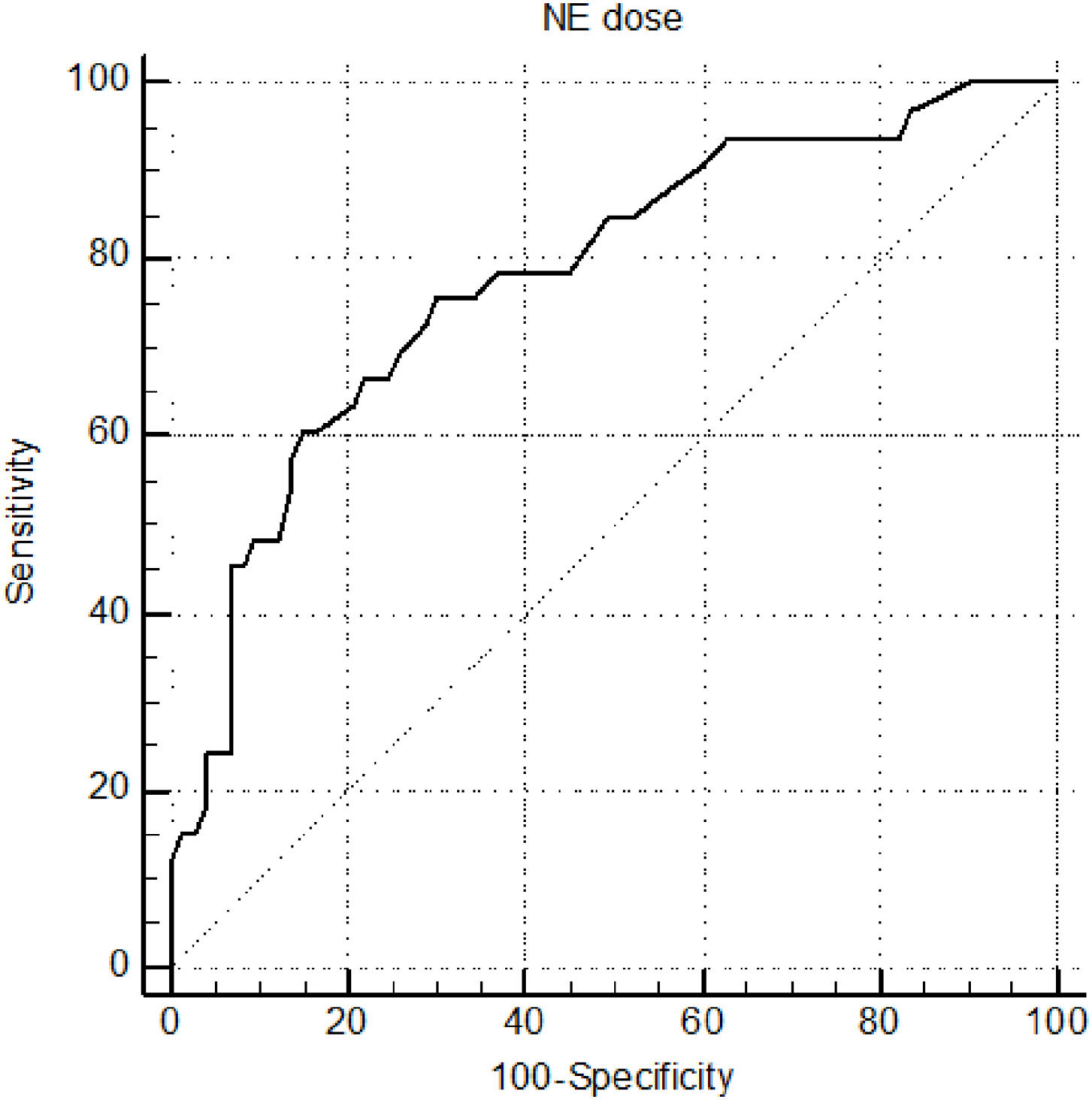
Receiver operating characteristic curve for NE dose for predicting 6-h lactate clearance.

The cutoff value and areas under the ROC curves for the related hemodynamic and metabolic variables used for predicting 6-h lactate clearance are shown in Table 3. The cutoff of NE was ≥ 0.32 μg·kg^−1^·min^−1^ for predicting 6-h lactate clearance after resuscitation, resulting in a sensitivity of 75.76% and a specificity of 70.0%, based on the maximum Youden index. The NE AUC_ROC_ was 0.782. The ROC curves for NE dose are shown in Figure 2. After resuscitation, in septic shock patients, there was significantly lower lactate clearance with high-dose NE than with low-dose NE (*p* < 0.001). Septic shock patients with high-dose NE had a significantly higher mortality rate than patients with low-dose NE (*p* < 0.001) (Table 4).

**Table 4:** Baseline characteristics and outcome between low and high NE dose groups.

	**NE dose < 0.32** **(*n* = 53)**	**NE dose ≥ 0.32 ** **(*n* = 53)**	**P Value**
Age, years	66.19 ± 11.81	61.21 ± 15.19	0.062
Sex (female/male)	34/19	32/21	0.689
APACHE II score	20.92 ± 5.24	21.06 ± 5.11	0.896
SOFA score	12.81 ± 3.08	12.57 ± 2.38	0.648
Heart rate (beats/min) 0 h	99.5 ± 19.6	100.0 ± 20.3	0.90
Heart rate (beats/min) 6 h	97.9 ± 18.0	99.5 ± 19.7	0.68
Temperature (°C) 0 h	37.5 (36.5–37.8)	37.0 (36.4–37.8)	0.18
Temperature (°C) 6 h	37.1 (36.7–37.8)	37.0 (36.5–38.0)	0.55
MAP (mmHg)0 h	86.1 ± 9.9	84.1 ± 8.4	0.27
MAP (mmHg)6 h	84.6 ± 10.8	83.5 ± 9.7	0.56
NE dosage (μg·kg^−1^·min^−1^) 0 h	0.20 (0.10–0.24)	0.55 (0.41–0.82)	0.001^*^
NE dosage (μg·kg^−1^·min^−1^) 6 h	0.20 (0.12–0.25)	0.60 (0.41–0.85)	0.001^*^
pH 0 h	7.36 ± 0.14	7.37 ± 0.13	0.48
pH 6 h	7.37 ± 0.11	7.38 ± 0.11	0.74
Base deficit, (mmol/L) 0 h	2.12 ± 7.02	2.76 ± 6.45	0.63
Base deficit, (mmol/L) 6 h	2.81 ± 5.35	2.69 ± 5.87	0.91
Lactate (mmol/L) 0 h	4.0 (3.3–4.9)	5.0 (3.3–6.7)	0.03^*^
Lactate (mmol/L) 6 h	2.8 (2.0–3.9)	4.2 (3.0–6.4)	0.001^*^
CVP (mmHg) 0 h	9.3 ± 2.1	9.5 ± 2.3	0.56
CVP (mmHg) 6 h	8.9 ± 2.1	9.1 ± 2.1	0.68
Pv-aCO_2_ (mmHg) 0 h	6.0 (4.0–8.1)	6.9 (4.5–9.2)	0.16
ScvO_2_ (%) 0 h	75.7 ± 4.1	76.6 ± 4.5	0.31
6 h Lactate Clearance (%)	0.28 ± 0.24	0.06 ± 0.25	0.001^*^
Hemoglobin (g/dL)	8.63 ± 1.76	8.71 ± 1.53	0.79
Creatinine (mmol/L)	112.0 (79.0–178.5)	99.0 (74.5–149.0)	0.43
Urine output 6 h (ml)	440 (270–640)	420 (260–600)	0.53
D-Dimer (ug/mL)	2.53 (0.67–5.65)	2.20 (0.94–3.62)	0.91
Prothrombin Time (secs)	16.3 (14.3–18.7)	16.6 (14.4–20.1)	0.26
Total bilirubin (mmol/L)	20.9 (16.1–33.8)	28.4 (16.9–38.8)	0.06
Platelet, (per mm^3^)	154,000 (102,000–216,500)	152000 (82,000–214,500)	0.68
Albumin, g/L	29.4 ± 5.7	29.9 ± 6.5	0.72
WBC (per mm^3^)	14.1 ± 4.6	16.0 ± 6.2	0.08
30-day mortality rate (%)	26.4	69.8	0.001^*^

*Date were presented by mean ± SD or median (P25,P75); NE, norepinephrine; APACHE II, Acute physiology and chronic health II; SOFA, Sequential Organ Failure Assessment; MAP, mean arteial pressure; CVP, central venous pressure; ScvO_2_, central venous oxygen saturation; Pv-aCO_2_, Central Venous-to-Arterial CO2 Difference; WBC, white blood cell*.

* *Statistically significant, p < 0.05*.

## Discussion

Our observations reveal that some patients with septic shock still had poor lactate clearance after resuscitation of systemic hemodynamics. After patients achieved complete resuscitation, systemic hemodynamic variables (MAP, ScvO_2_, Pv-aCO_2_) did not predict lactate clearance further. Norepinephrine dosage was closely related to lactate clearance after resuscitation in septic shock and had good sensitivity and specificity for predicting 6-h lactate clearance < 10%. Blood lactate clearance is a classic indicator for assessing shock resuscitation; for every 10% increase in lactate clearance in septic shock patients, mortality can be reduced by 11% (3). Several high-quality studies in recent years have confirmed that blood lactate levels are still the best prognostic predictors of septic shock (13, 14). A notable ProCESS RCT study in 2014 found that systemic hemodynamic targets achieved did not improve mortality in patients with septic shock (15), and a later systematic review with more samples came to the same conclusion (16). Some patients with septic shock still have hyperlactacidemia after 24 h, even after completion of circulation resuscitation, and still have a poor prognosis (6). Consistent with previous findings, this study found that MAP, ScvO_2_, and Pv-aCO_2_ could not predict lactate clearance after resuscitation. Norepinephrine dose can better predict lactate clearance, and a norepinephrine dose >0.32 μg·kg^−1^·min^−1^ predicted blood lactate clearance < 10%, with a sensitivity of 75.76% and specificity of 70.0% after 6 h.

Septic shock is the most common distributive shock, accounting for more than 90% of all cases of distributive shock (17). Vasoplegia is a pathological syndrome of decreased vascular tension, and its main clinical feature is the occurrence of hypotension with normal or increased cardiac output (18). Norepinephrine is the only first-line vasopressor recommended in the guidelines for the diagnosis and treatment of septic shock (19). Another important hemodynamic characteristic of septic shock is microcirculatory shock; that is, there is still insufficient tissue perfusion after major circulatory resuscitation (20). Vasoplegia and microcirculation shock occur at the same time in most cases, and vascular endothelial dysfunction and NO, IL-1, TNF-α and other cytokine effects may be the common cause of vascular regulation damage, though the specific mechanism is still unclear (18). Current popular assessment methods for microcirculation disorders are evaluated using sidestream dark field (SDF) techniques. The disadvantage is that this involves local monitoring, which cannot predict microcirculation well in other unmonitored organs. Norepinephrine dose is currently the most effective clinical indicator of the degree of vascular paralysis. After cardiac output recovery, the higher the dose of norepinephrine is, the higher the degree of vasoplegia. The higher the degree of vasoplegia, the harder it is to transport oxygen to the terminal tissue and the more difficult it is to recover oxygen metabolism. Therefore, the norepinephrine dose is still directly related to oxygen metabolism disorder after resuscitation of systemic hemodynamics.

The relationship between norepinephrine and prognosis in septic patients is not consistent. Service de Reanimation et al. found that the norepinephrine dose and the norepinephrine dose needed to maintain a target blood pressure greater than 6 μg·kg-1·min-1 were independent death risk factors for patients with septic shock (10). However, Yamamura et al.'s study (9) obtained the opposite conclusion: there was no difference in 28-day mortality between the high-dose and low-dose norepinephrine groups in septic shock patients. A careful reading of the literature indicates that the norepinephrine dose in the study was the 7-day cumulative amount after the start of septic shock treatment. This calculation also includes norepinephrine before resuscitation in septic shock. Before the completion of septic shock resuscitation, there is a state of insufficient flow, that is, low cardiac output, so that the cumulative norepinephrine dose does not represent the degree of vasoplegia in septic shock and cannot fully represent the severity of septic shock. In our study, after resuscitation, ScvO_2_ ≥ 70% indicated that there was not insufficient blood flow, and the dose of norepinephrine could represent the severity of vasoplegia.

Some limitations of our study need to be acknowledged. First, the retrospective and monocentric nature of the study may limit the external validity of the results. Second, the number of cases was small, and there was a possibility of bias. Finally, there were no microcirculation evaluations in this study. We want to perform more in-depth research on microcirculation and norepinephrine in the future.

## Conclusions

This study found that some patients with septic shock still had tissue oxygen metabolism disorder after achieving normal systemic hemodynamic and metabolic variables. A large dosage of norepinephrine is related to persistent non-recovery of tissue oxygen metabolism disorder. The dosage of norepinephrine might therefore be a valuable adjunct for identifying septic shock patients who have persistent tissue oxygen metabolism disorder after resuscitation.

## Data Availability Statement

The original contributions presented in the study are included in the article/Supplementary Material, further inquiries can be directed to the corresponding author/s.

## Ethics Statement

The authors are responsible for all aspects of the work in ensuring that questions related to the accuracy or integrity of any part of the work are appropriately investigated and resolved. The study was approved by the Ethical Committee of The First Affiliated Hospital of Anhui Medical University (the number/ID of the approval is PJ 2021-3-15). Written informed consent for participation was not required for this study in accordance with the national legislation and the institutional requirements.

## Author Contributions

CY and MS wrote the main manuscript text, included: contributed to the conception, designed the work, and analyzed and interpreted data. WF collected the data regarding the paper. MS approving data presentation as representative of the original data and foreseeing and minimizing obstacles to the sharing of data described in the work. All authors read and approved the final manuscript.

## Funding

This work was supported by Dr Ping Zhou for her critical revision of the manuscript.

## Conflict of Interest

The authors declare that the research was conducted in the absence of any commercial or financial relationships that could be construed as a potential conflict of interest.

## Publisher's Note

All claims expressed in this article are solely those of the authors and do not necessarily represent those of their affiliated organizations, or those of the publisher, the editors and the reviewers. Any product that may be evaluated in this article, or claim that may be made by its manufacturer, is not guaranteed or endorsed by the publisher.

## Abbreviations

ScvO_2_: Central Venous Oxygen Saturation
MAP: Mean Arterial Pressure
CVP: Central Venous Pressure
NE: Norepinephrine
ROC: Receiver Operating Characteristic
LC: Lactate Clearance
ICU: Intensive Care Unit
APACHE II: Acute Physiology and Chronic Health Evaluation II
SOFA: Organ Failure Assessment
Pv-aCO_2_: Central Venous-To-Arterial CO_2_ Difference
WBC: White Blood Cell
SDF: Sidestream Dark Field.
